# Four-Year Report of Iatrogenic Bile Duct Injury Repair from a Referral Hepatobiliary Center

**DOI:** 10.34172/mejdd.2024.385

**Published:** 2024-07-31

**Authors:** Seyed Yahya Zarghami, Roya Ghafoury, Nasir Fakhar, Fatemeh Afrashteh, Davod Tasa, Zeeshan Hyder

**Affiliations:** ^1^Department of General Surgery, School of Medicine, Firoozgar hospital, Iran University of Medical Sciences, Tehran, Iran; ^2^School of Medicine, Iran University of Medical Sciences, Tehran, Iran; ^3^Tehran University of Medical Sciences, Shariati Hospital, Tehran, Iran; ^4^Organ Transplantation Center, Masih Daneshvari Hospital, Shahid Beheshti University of Medical Sciences, Tehran, Iran; ^5^Dr.Ziauddin University Hospital, Karachi, Pakistan

**Keywords:** Hepatectomy, Iatrogenic bile duct injury, Bile duct repair, Hepatic artery injury, Hepatic ischemia

## Abstract

**Background:**

Iatrogenic bile duct injury (BDI) is one of the most common complications that challenge surgeons performing laparoscopic cholecystectomy (LC). As the number of LC surgeries increases, a pattern of BDI is emerging, but little is known about this matter. The purpose of this study was to assess the pattern of post-LC BDIs directed in a referral center in Iran.

**Methods:**

Post-BDI patients referred to a hepatobiliary center were studied. Demographic data, clinical status, diagnostic examinations, referral time, post-referral management, and morbidity were analyzed.

**Results:**

Nine out of 68 patients had Charcot’s cholangitis triad featuring right upper quadrant abdominal pain, fever, and icter. Fever, icter, and itching were the most frequent symptoms. Increased bilirubin, leukocytosis, and abnormal liver function test (LFT) were the most common laboratory abnormalities. 30 patients experienced hepatic artery injury. Out of them, six patients experienced hepatectomy due to hepatic ischemia. Two patients were re-admitted and re-operated, and four patients died.

**Conclusion:**

Early and correct treatment by a hepatobiliary surgeon experienced in the management of these types of injuries prevents further complications in patients suffering from iatrogenic BDI. Postoperative complications of bile duct repair, such as anastomosis stricture, are possible; thus, patients need long-term and thorough postoperative observation.

## Introduction

 Bile duct injury (BDI) can happen during numerous surgical operations such as hepatectomy, gastrectomy, or portocaval shunt, yet about 80-85% of such cases are related to bile duct surgeries, particularly cholecystectomy.^1^ Cholecystectomy is the most commonly performed elective operation on the abdomen (∼750 000 each year) in the United States of America. The removal of gallbladder has risen since the clinical presentation of laparoscopic cholecystectomy (LC) in 1989.^2^ Although LC is the optimal technique for gallbladder resection and has benefited patients with shorter lengths of hospital stay and less postoperative pain, it is related to a higher chance of BDI compared with open (traditional) cholecystectomy (from 0.1%-0.2% in open cholecystectomy to 0.4%-0.7% in LC).^3,4^ BDI occurrence during a cholecystectomy is affected by many factors, such as demographic factors, disease severity, and the quality of hospitals and experiences of surgeons.^5,6^ Furthermore, some studies have shown that using intraoperative cholangiography (IOC) could decrease the incidence and severity of BDI.^7,8^ Non-invasive methods such as endoscopic or percutaneous interventions can be used to treat minor BDI and biliary leaks with good results. However, procrastinating methods such as endoscopic or percutaneous stenting and drain placement followed by postponed surgical repair are usually required for major BDI after a few weeks to resolve local inflammation.^9,10^ It has been well established that iatrogenic BDI during LC affects both short-term outcomes, including longer hospitalization, increased hospital fees, and perioperative morbidity, and long-term outcomes, such as anastomotic stricture, secondary biliary disorders, and increased mortality.^11,12^ Surgical repair of BDI is known to be a technically challenging process that is best managed by qualified hepatobiliary surgeons. Data exist on commonly reported surgical outcomes by such surgeons, reported a low risk of operative mortality and high rates of short-term and intermediate-term success without the need for re-operation.^13^ This study was undertaken to determine factors affecting BDI repair outcomes in Iran.

## Materials and Methods

 This is an observational and retrospective study performed on 68 patients admitted to the Department of Hepatobiliary Surgery at Imam Khomeini Hospital, Iran University of Medical Sciences, Iran (a major referral center), from December 2016 to January 2020. All patients had iatrogenic BDI on admission, which was developed after a cholecystectomy surgery (open and laparoscopic) and underwent repair surgery by experienced hepatobiliary surgeons. The medical records of patients after repair surgery were studied and demographic data (such as age and sex), hospital length of stay, injury to repair surgery interval, presenting signs and symptoms, and laboratory data were extracted and then analyzed.

 SPSS software (version 16.0) was used for data collection and analyses. The distribution of continuous variables was determined utilizing the Kolmogorov-Smirnov test and were reported as mean and standard deviations or as median and inter-quartile range (IQR), as appropriate. A comparison of categorical variables was performed using the chi-square test or Fisher exact test. To assess the risk of post-referral complications, univariate and multivariate analysis was performed utilizing the chi-square test and binary logistic regression. A *P* value of less than 0.05 was considered statistically significant.

## Results

 Sixty-eight patients, 50 women, and 18 men, were included in this study. The mean age of participants was 46.44 ± 14.19 years. The mean duration of hospitalization was 5.68 ± 4.65 days. Although most of them (34 patients) had 0 to 5 days of hospitalization, four patients were hospitalized for 16 to 20 days. Laparatomic and laparoscopic cholecystectomy were performed for 23 and 45 patients, respectively.

 Injury to repair surgery interval was less than 2 weeks in 24 patients, 2-6 weeks in 22 patients, and more than 6 weeks in 22 patients. The most common surgery type was hepaticojejunostomy, which was performed in 59 patients. According to Bismuth classification, 21 injuries were Class 2, 20 patients had Class 4, 16 patients had Class 3, and others had other classes of injuries.

 Fever, icter, and itching were the most frequent symptoms. Nine patients (three men and six women) had Charcot’s cholangitis triad (right upper quadrant abdominal pain, fever, and icter). All patients underwent hepatojejunostomy surgery. The class of injury in these patients were class II, IV, and previous anastomotic stricture, and three patients were in each category. Less than 2 weeks, 2-6 weeks, and more than 6 weeks injury to surgery interval were seen in one, three, and five patients, respectively. Among patients with Charcot’s cholangitis triad, two patients experienced right hepatic artery injury, and one patient experienced hepatic ischemia and peritonitis. Two patients re-admitted and re-operated, and one patient died.

 Among 47 patients who had laboratory abnormalities, increased bilirubin, leukocytosis, and abnormal liver function test (LFT) were the most abnormal results. Overall, 30 (63.8%) patients had right hepatic artery injury, and in six (20%) patients, it caused hepatic ischemia, and all of them went through hepatectomy. Other hepatic artery injuries did not meet the criteria needed for hepatectomy. Complementary patient characteristics, including symptoms, lab data, and procedures, are summarized in Tables 1 and 2.

**Table 1:** Demographic data and surgical features of the patients

	**Numbers **	**Percent **
Hospitalization category		
0-5 days	34	50
6-10 days	16	23.5
11-15 days	14	20.6
16-20 days	4	5.9
Injury to surgery interval		
Less than 2 weeks	24	35.3
2-6 weeks	22	32.4
More than 6 weeks	22	32.4
Type of surgery		
Hepatectomy + hepaticojeujunostomy	6	8.8
Hepaticojeujunostomy	59	86.8
Primary repair	2	2.9
Choledochoduodenostomy	1	1.5
Injury Class		
Class 1	2	2.9
Class 2	21	30.9
Class 3	16	23.5
Class 4	20	29.4
Previous anastomotic stricture	7	10.3
Open cystic duct	1	1.5
Accessory duct	1	1.5

**Table 2:** Clinical and laboratory data of the patients

**Variables**	**Yes (N, %)**	**No (N, %)**
Bile leak	3, 4.4%	65, 95.6%
Biliary stricture	1, 1.5%	67, 98.5%
Collection during surgery	7, 10.3%	61, 89.7%
Anastomosis stricture	1, 1.5%	67, 98.5%
Post operation complication	21, 30.9%	47, 69.1%
Wound infection	15, 22.1%	53, 77.9%
Peritonitis	1, 1.5%	67, 98.5%
Collection peritonitis	9, 13.2%	59, 86.8%
Biloma	29, 42.6%	39, 57.4%
Hepatic artery injury	30, 44.1%	38, 55.9%
Hepatic ischemia	6, 8.8%	62, 91.2%
Re-operation	4, 5.9%	64, 94.1%
Percutaneous drainage	9, 13.2%	59, 86.8%
Re-admission	6, 8.8%	62, 91.2%
Deceased	4, 5.9%	64, 94.1%
Chest tube	3, 4.4%	65, 95.6%
Increased bilirubin	27, 39.7%	41, 60.3%
Increased INR	9, 13.2%	59, 86.8%
Abnormal LFT	15, 22.1%	53, 77.9%
Leukocytosis	16, 23.53%	52, 76.47%
Lab abnormality	47, 69.1%	21,30.9%
Fever	18, 26.5%	50, 73.5%
Icter	17, 25%	51, 75%
Itching	17, 25%	51, 75%
Previous surgery	31, 45.6%	37, 54.4%

INR: International normalized ratio, LFT: Liver function test.

 The duration of hospitalization between types of surgery was significantly different. This variance was seen among the hepatectomy + hepaticojejunostomy and hepaticojejunostomy group (P = 0.006): 13.1 ± 3.6 days in the hepatectomy + hepaticojejunostomy group vs 7 ± 4.5 days in the hepatojejunostomy group.

 Four patients, including one patient with less than 2 weeks of injury to surgery interval and three patients with 2-6 weeks, needed re-operation. Six patients were re-admitted (two hepatectomy + hepaticojejunostomy and four hepatojejunostomy), and there was no significant relation between the need for re-admission and types of surgery. Among these six patients, three patients had less than 2 weeks injury to surgery interval, two patients had 2-6 weeks, and one patient had more than 6 weeks. There was no significant relation between injury to surgery interval and re-admission or re-operation. Four patients died after the operation (one hepatectomy + hepatojejunostomy and three hepatojejunostomy). Among them, two patients had less than 2 weeks injury to surgery interval, and two patients had more than 6 weeks.

 Collection, peritonitis, fever, right hepatic artery injury, and biloma were seen during operation significantly different between injury classes, and it was more in Class 4 rather than other injuries (*P* = 0.004, 0.027, 0.001, and 0.002, respectively).

## Discussion

 After the first introduction and vast practice of LC in 1990s, the incidence of biliary injuries and related complications such as bile duct strictures has increased. The increase in the occurrence of major bile duct injuries was rather noticeable as the estimated occurrence of major bile duct injuries raised from 0.1% to 0.3% during the vast practice of open cholecystectomy to nearly 0.4% to 0.86% for LC.^14^

 A study by O’Brien, and colleagues demonstrated that the length of hospital stay due to an LC-related BDI is increased to approximately 8.6 days compared with 4.8 days for uncomplicated LC. Furthermore, biliary injuries at the time of LC can be twice the cost of an uneventful method, and the mortality rate is considerably high.^15^ In our study, the mean patients’ length of hospital stay with biliary injury was about 5.68 ± 4.65 days compared with about 2 days for an uneventful LC. Some literature suggests that the occurrence of acute cholecystitis during the surgery is a strong predisposing factor for LC-related biliary injuries.^16,17^ However, other studies reported no significant association.^18^ A systematic review consisting of 32 case-control studies disclosed that inflammation (at acute and sub-acute levels) at Calot’s triangle was a significant associated factor for biliary injury. Also, age (over 40 years), liver lab abnormalities prior to surgery, and variations in gallbladder anatomy were other risk factors.^17^ In our sample, nine patients (three men and six women) out of 68 patients (0.13%) had Charcot’s cholangitis triad, in which two patients experienced hepatic artery injury, and one patient developed hepatic ischemia.

 The level of injury is strongly connected with the occurrence of biliary stricture after reconstruction. One-third of surgeries with an injury proximal to the bifurcation of the biliary tree result in post-repair stricture.^19,20^ Among our patients, 21 injuries were Class 2, 20 patients had Class 4, 16 patients had Class 3, and others had other classes of injuries. Only one patient experienced anastomotic stricture, which had a Class 4 injury that is in accordance with previous studies.^19,20^

 Different factors affect the outcome of surgical treatment, including the form of reconstructive procedures, competence of the attending surgeons, extent of the damage, occurrence of proximal dilation, timing of the intervention after injury, and presence of local inflammation at the time of surgery.^21^ We found out that type of performed surgery affects the hospitalization duration significantly as the patients in the hepatojejunostomy group experienced fewer days of hospitalization (7 ± 4.5 days) while patients in the hepatectomy + hepatojejunostomy group had more days of hospital residing (13.1 ± 3.6 days, *P* = 0.006). Types of surgery had no significant relation with the need for re-admission.

 Several authors have reported concurrent vascular injuries that occurred in the presence of BDI, resulting in both instant and long-term management requirements.^22,23^ Considering the anatomical proximity of the common hepatic duct to the right hepatic artery, particularly at the confluence of hepatic ducts, concomitant arterial injury is rather difficult to avoid.^24^

 Truant and others demonstrated that there was a greater risk of hepatectomy for patients who developed simultaneous arterial and Strasberg E4 or E5 injuries, with an odds ratio of 43.3 (95% CI 8.0–234.2) compared with patients with neither of the mentioned injuries. According to their results, there were major disparities in the rates of combined vascular injuries and complex injuries between teams reporting hepatectomies carried out in the management of patients with BDI and those carrying out biliary repair solely by hepaticojejunostomy.^25^

 A survey of 74 patients with BDI indicated that the need for surgical intervention and its extension depends on the type of arterial injury and also the anatomical location of the injury, at or above the bifurcation of the hepatic duct.^25^ Another study further showed that patients with hepatic arterial injury who have undergone hepatobiliary repair by an inexperienced surgeon in such surgeries were at greater risk for hepatectomy compared with those without such injury.^26^ These studies highlight the importance of early referral to a tertiary center in patients with BDI.^27^

 In our study, 30 out of 68 patients (44.1%) experienced hepatic artery injury and in six patients (8.8%) it caused hepatic ischemia. Six patients out of 30 patients (20%) with hepatic artery injury underwent hepatectomy surgery, among which all of them had hepatic ischemia. Also, one hepatectomy surgery was performed on a patient without hepatic artery injury due to the severity of biliary tract damage. Our results are in accordance with the findings of Bektas and colleagues^26^ and Stewart et al,^27^ in which the experience of surgeons in hepatobiliary repair was a determinative factor.

## Conclusion

 Biliary tract surgical procedures are one of the most common surgeries performed worldwide. There has been a recent increase in the incidence of iatrogenic BDI, which has been connected with the increased practice of LC globally. In pursuance of reducing the risk of iatrogenic BDI throughout surgery, it is crucial to be precise in the appropriate visualization and identification of anatomical structures before transecting or ligating the surgical area.^28^ Following biliary injury development, immediate recognition and proper treatment are vital. Patients’ clinical status, such as Charcot’s cholangitis triad, biloma, peritonitis, etc, is a determinant factor for patient prognosis; thus, spotting them and treatment initiation must be emphasized. It is essential to keep in mind that following BDI, surgeons refer the patients to secondary centers for drainage placement and then to tertiary centers for final repair. Postoperative complications of bile duct repair, such as anastomosis stricture, are possible; thus, patients need long-term and thorough postoperative observation.

## References

[1] Sharma S, Behari A, Shukla R, Dasari M, Kapoor VK (2020). Bile duct injury during laparoscopic cholecystectomy: an Indian e-survey. Ann Hepatobiliary Pancreat Surg.

[2] Kapoor VK. Epidemiology of bile duct injury. In: Kapoor VK, ed. Post-cholecystectomy Bile Duct Injury. Singapore: Springer; 2020. p. 11-9. 10.1007/978-981-15-1236-0_2.

[3] Brunt LM, Deziel DJ, Telem DA, Strasberg SM, Aggarwal R, Asbun H (2020). Safe Cholecystectomy Multi-society Practice Guideline and State of the Art Consensus Conference on Prevention of Bile Duct Injury During Cholecystectomy. Ann Surg.

[4] Nuzzo G, Giuliante F, Giovannini I, Ardito F, D’Acapito F, Vellone M (2005). Bile duct injury during laparoscopic cholecystectomy: results of an Italian national survey on 56 591 cholecystectomies. Arch Surg.

[5] Viste A, Horn A, Øvrebø K, Christensen B, Angelsen JH, Hoem D (2015). Bile duct injuries following laparoscopic cholecystectomy. Scand J Surg.

[6] Krähenbühl L, Sclabas G, Wente MN, Schäfer M, Schlumpf R, Büchler MW (2001). Incidence, risk factors, and prevention of biliary tract injuries during laparoscopic cholecystectomy in Switzerland. World J Surg.

[7] Rystedt JML, Wiss J, Adolfsson J, Enochsson L, Hallerbäck B, Johansson P (2021). Routine versus selective intraoperative cholangiography during cholecystectomy: systematic review, meta-analysis and health economic model analysis of iatrogenic bile duct injury. BJS Open.

[8] Christou N, Roux-David A, Naumann DN, Bouvier S, Rivaille T, Derbal S (2021). Bile duct injury during cholecystectomy: necessity to learn how to do and interpret intraoperative cholangiography. Front Med (Lausanne).

[9] Ceresoli M, Coccolini F, Biffl WL, Sartelli M, Ansaloni L, Moore EE (2020). WSES guidelines updates. World J Emerg Surg.

[10] Lau WY, Lai EC, Lau SH (2010). Management of bile duct injury after laparoscopic cholecystectomy: a review. ANZ J Surg.

[11] Booij KAC, de Reuver PR, van Dieren S, van Delden OM, Rauws EA, Busch OR (2018). Long-term impact of bile duct injury on morbidity, mortality, quality of life, and work-related limitations. Ann Surg.

[12] Koppatz H, Sallinen V, Mäkisalo H, Nordin A (2021). Outcomes and quality of life after major bile duct injury in long-term follow-up. Surg Endosc.

[13] Fletcher R, Cortina CS, Kornfield H, Varelas A, Li R, Veenstra B (2020). Bile duct injuries: a contemporary survey of surgeon attitudes and experiences. Surg Endosc.

[14] Hogan NM, Dorcaratto D, Hogan AM, Nasirawan F, McEntee P, Maguire D (2016). Iatrogenic common bile duct injuries: increasing complexity in the laparoscopic era: a prospective cohort study. Int J Surg.

[15] O’Brien S, Wei D, Bhutiani N, Rao MK, Johnston SS, Patkar A (2020). Adverse outcomes and short-term cost implications of bile duct injury during cholecystectomy. Surg Endosc.

[16] Romashchenko PN, Maistrenko NA, Pryadko AS, Aliev AK, Aliev RK, Zherebtsov ES (2020). Prevention and treatment bile ducts injuries in patients with acute cholecystitis. Ann HPB Surg.

[17] Yang S, Hu S, Gu X, Zhang X (2022). Analysis of risk factors for bile duct injury in laparoscopic cholecystectomy in China: a systematic review and meta-analysis. Medicine (Baltimore).

[18] Ooi LL, Goh YC, Chew SP, Tay KH, Foo E, Low CH (1999). Bile duct injuries during laparoscopic cholecystectomy: a collective experience of four teaching hospitals and results of repair. Aust N Z J Surg.

[19] Pandit N, Yadav TN, Awale L, Deo KB, Dhakal Y, Adhikary S (2020). Current scenario of post-cholecystectomy bile leak and bile duct injury at a tertiary care referral centre of Nepal. Minim Invasive Surg.

[20] Mercado MA, Chan C, Orozco H, Hinojosa CA, Podgaetz E, Ramos-Gallardo G (2005). Prognostic implications of preserved bile duct confluence after iatrogenic injury. Hepatogastroenterology.

[21] Archer SB, Brown DW, Smith CD, Branum GD, Hunter JG (2001). Bile duct injury during laparoscopic cholecystectomy: results of a national survey. Ann Surg.

[22] Li J, Frilling A, Nadalin S, Paul A, Malagò M, Broelsch CE (2008). Management of concomitant hepatic artery injury in patients with iatrogenic major bile duct injury after laparoscopic cholecystectomy. Br J Surg.

[23] Renz BW, Bösch F, Angele MK (2017). Bile duct injury after cholecystectomy: surgical therapy. Visc Med.

[24] Pesce A, Palmucci S, La Greca G, Puleo S (2019). Iatrogenic bile duct injury: impact and management challenges. Clin Exp Gastroenterol.

[25] Truant S, Boleslawski E, Lebuffe G, Sergent G, Pruvot FR (2010). Hepatic resection for post-cholecystectomy bile duct injuries: a literature review. HPB (Oxford).

[26] Bektas H, Schrem H, Winny M, Klempnauer J (2007). Surgical treatment and outcome of iatrogenic bile duct lesions after cholecystectomy and the impact of different clinical classification systems. Br J Surg.

[27] Stewart L, Robinson TN, Lee CM, Liu K, Whang K, Way LW (2004). Right hepatic artery injury associated with laparoscopic bile duct injury: incidence, mechanism, and consequences. J Gastrointest Surg.

[28] de Reuver PR, Grossmann I, Busch OR, Obertop H, van Gulik TM, Gouma DJ (2007). Referral pattern and timing of repair are risk factors for complications after reconstructive surgery for bile duct injury. Ann Surg.

